# Fractional Synthesis Rates of Individual Proteins in Rat Soleus and Plantaris Muscles

**DOI:** 10.3390/proteomes8020010

**Published:** 2020-05-11

**Authors:** Connor A. Stead, Stuart J. Hesketh, Samuel Bennett, Hazel Sutherland, Jonathan C. Jarvis, Paulo J. Lisboa, Jatin G. Burniston

**Affiliations:** 1 Research Institute for Sport & Exercise Sciences, Liverpool John Moores University, Liverpool L3 3AF, UK; C.Stead1@2016.ljmu.ac.uk (C.A.S.); S.J.Hesketh@2014.ljmu.ac.uk (S.J.H.); S.Bennett@2014.ljmu.ac.uk (S.B.); H.Sutherland@ljmu.ac.uk (H.S.); J.C.Jarvis@ljmu.ac.uk (J.C.J.); 2 Department of Applied Mathematics, Liverpool John Moores University, Liverpool L3 3AF, UK; P.J.Lisboa@ljmu.ac.uk

**Keywords:** deuterium oxide, fractional synthesis rate, stable isotope labelling, skeletal muscle, protein synthesis, protein turnover, dynamic proteome profiling

## Abstract

Differences in the protein composition of fast- and slow-twitch muscle may be maintained by different rates of protein turnover. We investigated protein turnover rates in slow-twitch soleus and fast-twitch plantaris of male Wistar rats (body weight 412 ± 69 g). Animals were assigned to four groups (*n* = 3, in each), including a control group (0 d) and three groups that received deuterium oxide (D_2_O) for either 10 days, 20 days or 30 days. D_2_O administration was initiated by an intraperitoneal injection of 20 μL of 99% D_2_O-saline per g body weight, and maintained by provision of 4% (*v*/*v*) D_2_O in the drinking water available ad libitum. Soluble proteins from harvested muscles were analysed by liquid chromatography–tandem mass spectrometry and identified against the SwissProt database. The enrichment of D_2_O and rate constant (*k*) of protein synthesis was calculated from the abundance of peptide mass isotopomers. The fractional synthesis rate (FSR) of 44 proteins in soleus and 34 proteins in plantaris spanned from 0.58%/day (CO1A1: Collagen alpha-1 chain) to 5.40%/day NDRG2 (N-myc downstream-regulated gene 2 protein). Eight out of 18 proteins identified in both muscles had a different FSR in soleus than in plantaris (*p* < 0.05).

## 1. Introduction

Skeletal muscle exhibits a broad phenotypic range depending on its anatomical location and function within each organism. The heterogeneity in muscle phenotype is underpinned by differences in the proportion of fast- and slow-twitch fibres within a muscle. The contractile and metabolic properties of different muscles and fibre types have been studied and extensively reviewed in [1]. Differences in fibre type are associated with differences in the relative content of myosin heavy chain (MyHC) isoforms, which are accepted as being the primary marker of fibre type [2]. Omic techniques have provided more comprehensive data and comparative analysis of protein abundance profiles of striated muscles [3] was among the first work reported in muscle proteomics. Bottom-up proteomic methods later allowed a broader survey of differences between archetypal fast- and slow-twitch muscle in mice [4]. Consistent with earlier bio- and histo-chemical studies (e.g., [5,6]), the proteome analyses report enrichment of metabolic enzymes associated with the characteristic substrate preferences of fast and slow muscle phenotypes. Subunits of ATP synthases and other components of the mitochondrial respiratory chain are enriched in slow-oxidative muscle [7], whereas enzymes of high-energy phosphate metabolism (e.g., creatine kinase) and glycolysis are dominant in fast-glycolytic fibre types [3,7]. A recent iteration on this theme reported proteome profiles of single fibres extracted from human muscle classified by their dominant MyHC isoform [8]. Such analyses provide the fundamental basis for physiological genomic studies aiming to establish links between the proteome and muscle function. 

Protein abundance is the net product of the processes of synthesis and degradation, termed protein turnover. In the absence of cellular or mechanical perturbation (i.e., in resting muscle) protein abundance is stable and it is assumed synthesis is equal to degradation. Therefore, the terms of protein synthesis and protein turnover may be used interchangeably. The characteristic differences in protein abundance between fast- and slow-twitch muscle may be the result of differences in protein synthesis, which can be studied in vivo via biosynthetic labelling using radio- or stable-isotope-labelled amino acids. Comprehensive analyses of protein synthesis during developmental growth of rats [9] established a paradigm of greater turnover in slow- compared to fast-twitch muscle. This relationship was evident throughout post-natal growth and maturation of the rat and was attributed to differences in the activation pattern between slow-twitch postural muscle and fast-twitch locomotors. Muscle contraction increases protein synthesis rates in muscle [10], therefore, the greater frequency of recruitment of slow-twitch fibres may explain the greater turnover of protein in slow-twitch muscle versus more intermittent activation of fast muscle fibres.

Averaged data on the turnover of all muscle proteins (e.g., [9]) overlooks the broad range of different turnover rates exhibited by individual proteins demonstrated in yeast [11], mammalian cells [12], rodents [13] and human muscle [14]. Differences in turnover rate between slow and fast muscle phenotypes may be explained by the different protein compositions of fast and slow muscle fibres as well as their habitual patterns of activation. In addition to isoforms of MyHC, myofiber phenotype is characterised by slow and fast isoforms of ancillary proteins, including myosin light chains and subunits of the troponin complex. By contrast, the majority of metabolic enzymes are common to both fast and slow myofibers, albeit with different levels of abundance that complement the energetic demands of the myofibrillar contractile units. Targeted analysis of key muscle proteins brought the first insight to the fractional synthesis rate (FSR) of individual proteins labelled via intravenous infusion of a stable-isotope-labelled amino acid in vivo [15]. Based on gas chromatography mass spectrometry analyses of isotope enrichments in free and protein-bound amino acids, the FSR of MyHC (0.037%/h) was significantly different from the average turnover rate of mixed muscle proteins (0.047%/h) in human muscle [16]. Infusion of stable isotope-labelled amino acids constrains studies to a relatively short duration (e.g., min–h timescales), whereas the turnover of abundant tissue proteins in vivo occurs over a period of days. Longer-term labelling studies have been conducted in animals fed a diet enriched with a stable-isotope-labelled amino acid [13,17], whereas we have focused on the application of deuterium oxide (D_2_O or ^2^H_2_O) or “heavy water” [14,18,19,20,21]. D_2_O can be administered via the drinking water in free-living animals and the majority of amino acids are labelled intracellularly via transamination reactions [22]. When combined with peptide mass spectrometry, D_2_O labelling can provide information on enrichment of the precursor pool and FSR data for individual proteins [23] via mass isotopomer distribution analysis (MIDA). 

We recently used two-dimensional gel separation and peptide mass spectrometry to report synthesis data for eight proteins in four different striated muscles of the rat [18]. The FSR (%/day) of alpha-actin spanned an ~4-fold range from ~0.8 in fast-twitch extensor digitorum longus (EDL) to ~2.4 in the diaphragm and ~3.4 in the heart [18]. However, it was challenging to derive rate constant data for all proteins studied using MIDA. In most cases, we reported the total proportion of protein synthesised after 14 days rather than rate constants of protein-specific synthesis. This approach is consistent with equivalent work [24] but the ability to generate robust FSR data for individual proteins would facilitate better cross-comparison of data between studies. In the current work, we aimed to verify differences in protein-specific FSR in slow and fast muscle using bottom-up proteomics and we performed semi-log plot analysis over a time series of peptide mass isotopomer data rather than MIDA calculations. Deuterium incorporation into newly synthesised protein results in a decrease in the fractional abundance of the peptide monoisotopic peak, which follows a first-order exponential decay reflecting the incorporation of deuterium into the protein pool. The use of a time series experiment allows non-linear changes in D_2_O incorporation to be observed. Herein, data were analysed by semi-log plot and peptides with poor fitting (R_2_ < 0.85) data were excluded from further analysis, consistent with our recent work [19]. 

## 2. Materials and Methods

All experimental procedures were conducted under the British Home Office Animals (Scientific Procedures) Act 1986. Male Wistar rats (body weight 412 ± 69 g) were bred in-house in a conventional colony, housed in controlled conditions of 20 °C, 45% relative humidity and a 12 h light (600–1800 h) and 12 h dark cycle, with water and food available ad libitum. Animals were assigned to four groups (*n* = 3 in each), including a control group (0 days) and three groups (10, 20 and 30 days) that received deuterium oxide (^2^H_2_O or D_2_O; Sigma-Aldrich, St. Louis, MO, USA) administration that was initiated by an intraperitoneal loading injection of 20 μL of 99% D_2_O-saline per g body weight, and was then maintained by the administration of 4% (*v*/*v*) D_2_O in the drinking water available to the rats, which was topped up daily, consistent with our previous work [18].

At each time point, a group of animals was killed humanely in a rising concentration of CO_2_ followed by cervical dislocation and the plantaris and soleus muscles from the right hindlimb were isolated. Each muscle was cleaned of fat and connective tissue then weighed before being frozen in liquid nitrogen and stored at −80 °C pending further analysis. Muscles were ground under liquid nitrogen and a portion (~100 mg) homogenised on ice in 10 volumes of 7 M urea, 2 M thiourea, 4% (*w*/*v*) CHAPS, 40 mM Tris pH 7.4 including phosphatase inhibitor and complete protease inhibitor cocktails (Roche, Indianapolis, IN, USA). After centrifugation at 12,000× *g*, 4 °C for 45 min the supernatant was decanted and the protein concentration of a 5 μL aliquot measured by Bradford assay (Sigma, Poole, Dorset, UK).

Soluble proteins were processed for mass spectrometry analysis by in-solution digestion according to previous work from our laboratory [19]. Briefly, lysates containing 200 μg of protein were precipitated in 5 volumes of acetone at −20 °C overnight and then resuspended in UA buffer (8 M urea in 0.1 M Tris-HCl, pH 8.5). Samples were incubated at 37 °C for 15 min in UA buffer with 100 mM dithiothreitol (DTT) followed by 20 min at 4 °C in UA buffer containing 50 mM iodoacetamide (protected from light). Samples were washed twice with 100 μL UA buffer and transferred to 50 mM ammonium hydrogen bicarbonate (Ambic). Sequencing grade trypsin (Promega; Madison, WI, USA) in 50 mM Ambic was added at an enzyme to protein ratio of 1:50 and the samples were digested overnight at 37 °C. To terminate digestion, peptides were collected in 50 mM Ambic and trifluoracetic acid (TFA) was added to a final concentration of 0.2% (*v*/*v*). 

Digests containing 4 µg of peptides were de-salted using C_18_ Zip-tips (MerkMillipore, Darmstadt, Germany) and analysed by LC-MS consisting of nanoscale reverse-phase ultra-performance LC (NanoAcquity; Waters Corp., Milford, MA, USA) and online ESI QTOF MS/MS (Q-TOF Premier; Waters Corp.). Samples (5 μL corresponding to 1 μg tryptic peptides) were loaded by partial-loop injection on to a 180 μm ID × 20 mm long 100 Å, 5 µm BEH C_18_ Symmetry trap column (Waters Corp.) at a flow rate of 5 μL/min for 3 min in 2.5% (*v*/*v*) ACN, 0.1% (*v*/*v*) FA. Separation was conducted at 35 °C via a 75 μm ID × 250 mm long 130 Å, 1.7 µm BEH C_18_ analytical reverse-phase column (Waters Corp.). Peptides were eluted using a linear gradient that rose to 37.5% ACN 0.1% (*v*/*v*) FA over 60 min at a flow rate of 300 nL/min. Eluted peptides were sprayed directly into the MS via a NanoLock Spray source and Picotip emitter (New Objective, Woburn, MA, USA). Additionally, a LockMass reference (100 fmol/μL Glu-1-fibrinopeptide B) was delivered to the NanoLock Spray source of the MS and was sampled at 240 s intervals. For all measurements, the MS was operated in positive ESI mode at a resolution of 10,000 FWHM. Before analysis, the TOF analyser was calibrated using fragment ions of [Glu-1]-fibrinopeptide B from *m*/*z* 50 to 1990. Peptide MS were recorded between 350 and 1600 *m*/*z* and muscle samples were analysed in a randomised order interspersed by inter-sample blanks (5 μL 0.1% FA separated over a 15 min linear gradient). Data-dependent MS/MS spectra were collected from baseline (day 0) samples over the range 50–2000 *m*/*z*. The 5 most abundant precursor ions of charge 2+ 3+ or 4+ were selected for fragmentation using an elevated (20–40 eV) collision energy. A 30-s dynamic exclusion window was used to avoid repeated selection of peptides for MS/MS.

Soleus and plantaris data were analysed in separate Progenesis QI (Nonlinear Dynamics, Newcastle, UK) experiments, as described previously [14,19]. Analytical data were LockMass corrected using the doubly charged monoisotopic ion of the Glu-1-fibrinopeptide B and prominent ion features were used as vectors to warp each data set to a common reference chromatogram. An analysis window of 15–75 min and 350–1500 *m*/*z* was selected, and MS/MS spectra were searched against the Swiss-Prot database restricted to Rattus (8071 sequences) using a locally implemented Mascot server (v.2.2.03). The enzyme specificity was trypsin allowing 1 missed cleavage, carbamidomethyl modification of cysteine (fixed), deamidation of asparagine and glutamine (variable), oxidation of methionine (variable) and an *m*/*z* error of ± 0.3 Da. The Mascot output (XML format), restricted to non-homologous protein identifications was recombined with MS profile data in Progenesis. Peptide features with MOWSE scores < 30 (MudPIT scoring) were excluded. Peptide mass isotopomer abundance data were extracted from MS only spectra. 

The abundances (arbitrary units; AU) of the monoisotopic peak (m_0_), m_1_, m_2,_ m_3_ and m_4_ mass isotopomers were collected over the entire chromatographic peak for each unique peptide. Precursor enrichment was back-calculated from peptide mass isotopomer data according to [18]. Briefly, the enriched molar fraction of each mass isotopomer was calculated by subtracting the molar fraction of the unlabelled control peptide from the equivalent D_2_O-labelled peptide and the enrichment ratio between m_2_ and m_1_ mass isotopomers was used to calculate precursor enrichment (*p*) using:

(1) $$p=\left(\left(\frac{EM_{2}}{EM_{1}}\right)/\frac{\left(d-1\right)}{2}\right)\cdot 100$$

where *EM*_1_ is the enriched molar fraction of *m*_1_ and *EM*_2_ is the enriched molar fraction of *m*_2_ and *d* is the number of H-D exchange sites counted by referencing the peptide amino acid sequence against standard tables [25]. The median precursor enrichment was derived from the peptides belonging to serum albumin (ALBU) and this value of *p* was then used in Equation (4) to calculate the fractional rate of synthesis (FSR) of individual peptides.

Incorporation of ^2^H into newly synthesised protein in vivo results in a decrease in the molar fraction (*fm*_0_) of the monoisotopic (*m*_0_) peak [21]

(2) $$fm_{0}=\frac{m_{0}}{m_{0}+m_{1}+m_{2}+m_{3}\ldots }$$

The rate constant (*k*) for the decay of *fm*_0_ was calculated using semi-log plots to fit a linear model (*y* = *k*•*x* + *c*) to the change (Δ) in log-transformed *fm*_0_ data as a function of time.

(3) $$k=\frac{\Delta (-\ln(fm_{0}))-c}{\Delta \left(time\right)}$$

Data were filtered to exclude peptides with R-squared (R^2^) > 0.85 and FSR was derived by dividing *k* by the molar percent enrichment of deuterium in the precursor (*p*) pool and the number (*d*) of ^2^H exchangeable H—C bonds in each peptide.

(4) $$FSR=\frac{k}{\left(d\cdot p\right)}$$

Protein FSR was reported as the median values from unique peptides assigned to each protein (decimal values were multiplied by 100 to give FSR in %/day) in each animal. 

Statistical analyses were performed in R software for Statistical Computing. Differences in protein-specific FSR between plantaris and soleus were investigated by paired *t*-tests and *p*-values were corrected using the Benjamini–Hochberg formula. 

## 3. Results

**Figure 1:**
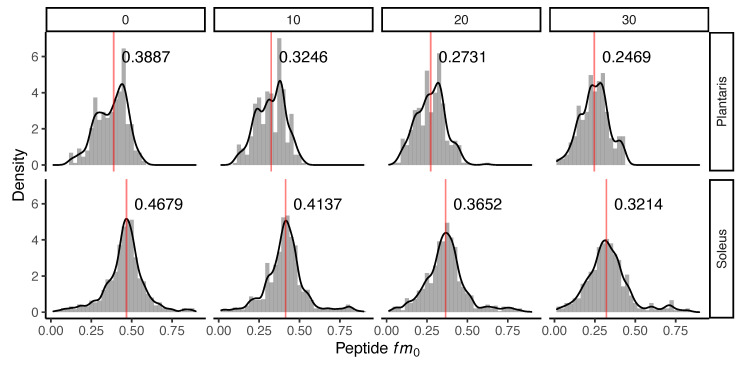
Incorporation of deuterium oxide in rat muscle in vivo. Histograms illustrating changes to the distribution of the fraction of the monoisotopic peak (*fm*_0_) of 240 peptides quantified in *n* = 3 rats at each experimental time point. Panels (left to right) represent data from control (day 0) rats that did not receive deuterium oxide (D_2_O), and independent groups of rats that received D_2_O for either 10 days, 20 days or 30 days duration. The median (red line) *fm*_0_ of peptides is reported in each panel for plantaris (top) and soleus (bottom). The incorporation of D_2_O into the protein pool in vivo resulted in a decline in *fm*_0_, evident as a time-dependent leftward shift in *fm*_0_ distribution.

**Figure 2:**
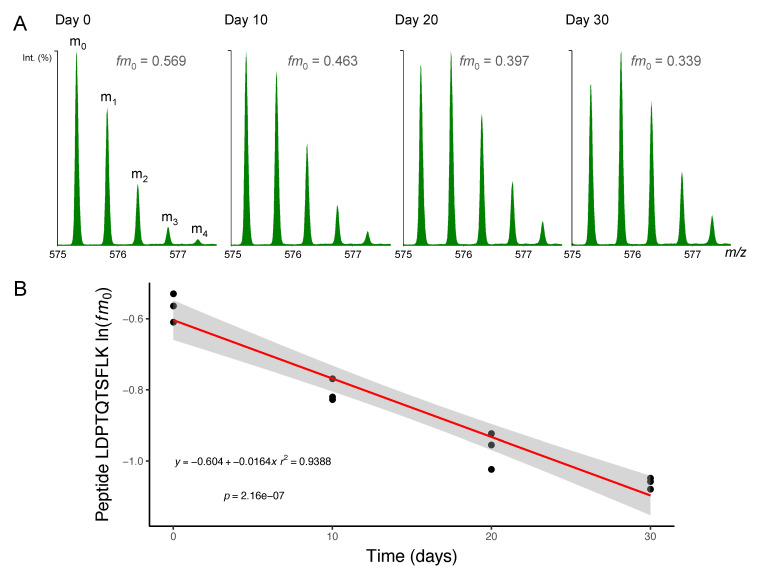
Mass spectrometry of deuterium-labelled peptides. Peptides were separated by nanoscale ultra-performance liquid chromatography and mass resolved as a series of mass isotopomers (*m*_0_, *m*_1_, *m*_2_, *m*_3_ and *m*_4_) using electrospray ionisation tandem mass spectrometry (ESI-MS/MS). (**A**) Mass spectra from peptide [M+2H]^2+^ 575.2995 *m/z* LDPTQTSFLK (residues 278–287) of protein NDRG2 (N-myc downstream-regulated gene 2 protein) are displayed from soleus muscle taken after 0, 10, 20 or 30 days of deuterium oxide (D_2_O) administration in vivo. The fraction of the monoisotopic peak (*fm*_0_) declines as a function of D_2_O incorporation into the protein pool over time. (**B**) Semi-log plot of *fm*_0_ data from peptide LDPTQTSKLK in *n* = 3 animals at each experimental time point. The slope of a linear model (red line; grey shaded area = 95% confidence interval) fitted to ln(*fm*_0_) data was used to calculate fractional synthesis rate (FSR) using Equations (3) and (4) (Materials and Methods).

Administration of D_2_O in vivo resulted in time-dependent changes to the peptide mass isotopomer pattern of plantaris and soleus muscle proteins (Figure 1). Biosynthetic labelling of muscle proteins was evident from the leftward shift in the distribution of the fractional abundance of peptide monoisotopic peaks (*fm*_0_). FSR data were calculated from time-dependent changes in peptide *fm*_0_ using semi-log plots (Figure 2). 

**Figure 3:**
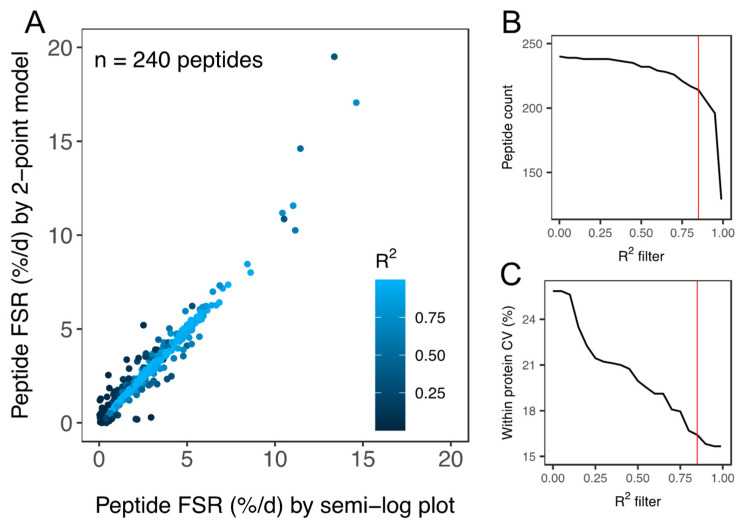
Quality assessment of peptide FSR data. (**A**) Scatter plot of peptide FSR (%/day) calculated by either a linear model fitted to peptide ln(*fm*_0_) data (i.e., semi-log plot method) at 0 days, 10 days, 20 days and 30 day or by a 2-point model fitted to peptide ln(*fm*_0_) data at 0 days and 30 days. Data points are coloured according to the goodness-of-fit (R^2^) to the linear model plotted using 4 time points (i.e., semi-log plot method). Filtering of peptide data assessed based on R^2^ was used to exclude peptides (**B**) with a poor fit to the expected linear model. Application of the R^2^ filter (**C**) decreased (improved) the coefficient of variation (CV) amongst peptides matched to the same protein within each animal. All peptides used in the onward analysis of protein FSR surpassed the quality threshold of R^2^ > 0.85 (red line in (**B**,**C**)).

Two-hundred and forty peptides were detected and peptide *fm*_0_ data were filtered based on goodness-of-fit (R^2^) to the expected linear model (Figure 3). Filtering of peptide data reduced the coefficient of variation (CV) of FSR data amongst peptides matched to an individual protein within each animal from 25.9% to 15.8% (Figure 3C). A quality threshold of R^2^ > 0.85 was applied and 214 peptides (within-protein CV < 16.7%) were used in the onward analysis of protein FSR. Precursor enrichment calculated from ALBU peptides was 2.3% ± 0.4%. FSR was calculated for 44 proteins in soleus and 34 proteins in plantaris (Table 1). The number of peptides analysed per protein spanned from 1–12. The average number of peptides per protein was 2.51 ± 2.05 and 51% (40 of 78) of proteins reported had one unique peptide that met the quality control criteria: (i) goodness-of-fit to the linear model of R^2^ > 0.85, (ii) detected in all (*n* = 3) animals at all experimental time points (0 day, 10 day, 20 day and 30 day). The R^2^ > 0.85 threshold excluded peptides with relatively high (e.g., >10%/day) turnover rates but did not significantly affect protein FSR values or conclusions arising from the comparison of soleus and plantaris muscle. Supplementary Table S1 reports the non-filtered list of peptide FSR and R^2^ data in the soleus and plantaris muscle of each animal.

**Figure 4:**
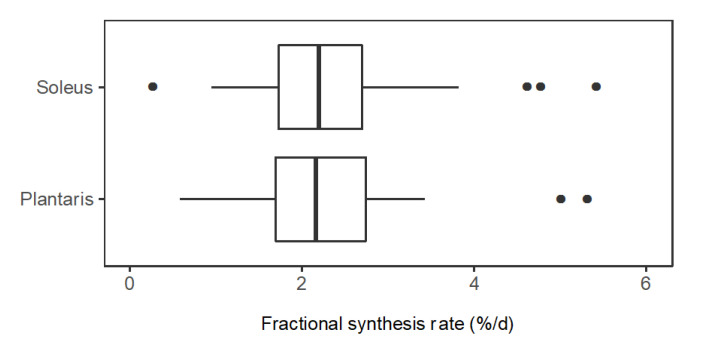
Distribution of protein fractional synthesis rates in slow- and fast-twitch muscle. Box and whisker plots of the fractional synthesis rates (FSR, %/day) of individual proteins within soleus muscle (*n* = 44 proteins) and plantaris muscle (*n* = 34 proteins) of rats (*n* = 3).

In slow-twitch soleus, FSR (%/day) ranged from 0.58 (CO1A1: Collagen α-1 (I) chain) to 5.40 (NDRG2: Protein NDRG2). The median (M) FSR was 2.26%/day, the lower quartile (Q_1_) was 1.71%/day and the upper quartile (Q_3_) was 2.77%/day. In fast-twitch plantaris muscle, FSR (%/day) ranged from 0.76 (H2B1: Histone type 2B type 1-α) to 5.00 (KCRS: Creatine kinase S-type). M = 2.26%/day, Q_1_ = 1.77%/day and Q_3_ = 2.58%/day (Figure 4). A paired *t*-test comparing mean FSR of mixed proteins in soleus (2.42%/day ± 1.03%/day) and plantaris (2.22%/day ± 0.82%/day) found no statistical difference (*p* = 0.117) between the muscles. 

**Figure 5:**
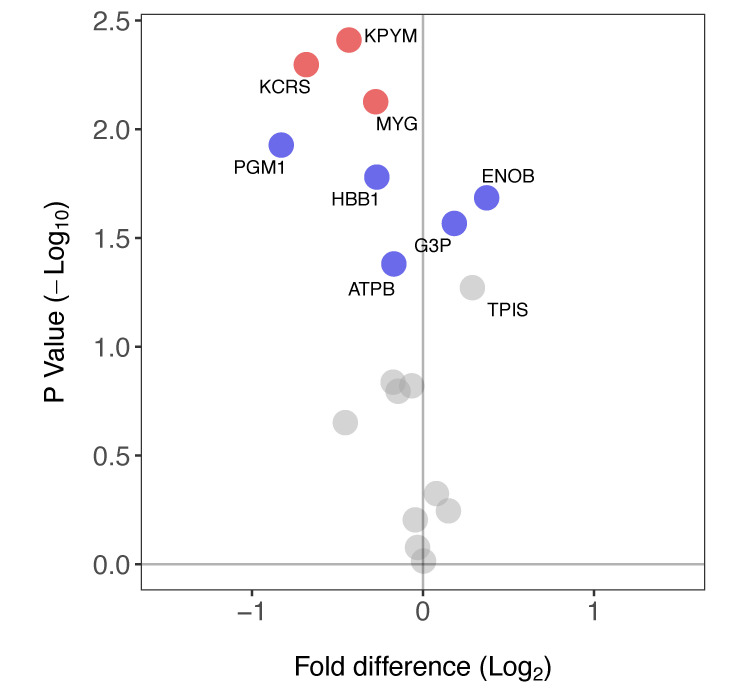
Differences in protein-specific synthesis rates in soleus versus plantaris muscle. Volcano plot reporting the difference in FSR in soleus compared to plantaris muscle. Paired *t*-tests were used to determine statistically different FSR of proteins between muscles (*n* = 3 in each group). Data are presented as a comparison of soleus versus plantaris: proteins with a significantly greater FSR (%/day) in soleus muscle have a positive fold-difference whereas those with a significantly greater FSR in the plantaris have a negative fold-difference. Proteins that had a significant difference in FSR are highlighted in blue (*p* < 0.05) or red (*p* < 0.05, BH-corrected). FSR values for each protein are reported in Table 1.

Eighteen proteins (Table 1) were identified in both soleus and plantaris samples. The FSR of eight of these proteins was significantly different between the soleus and plantaris muscles (Figure 5). Three proteins (ENOB, G3P, TPIS) primarily associated with glycolytic metabolism had a greater FSR in soleus than plantaris. In contrast, six proteins including mitochondrial as well as glycolytic enzymes (KPYM, KCRS, MYG, PGM1, HBB1 and ATPB) had a greater FSR in plantaris. 

## 4. Discussion

We have used stable isotope labelling in vivo and peptide mass spectrometry to report novel data on the turnover of individual proteins in slow- and fast-twitch rat muscle. When averaged, the turnover of mixed proteins surveyed in this experiment was not different between fast and slow muscle phenotypes (Figure 4). However, the turnover of numerous individual proteins was significantly different (Figure 5). Proteins that might be associated with fast-twitch skeletal muscle, such as glycolytic enzymes, had greater rates of turnover in slow-twitch soleus. Conversely, proteins typical of slow-oxidative muscle, such as myoglobin, had greater rates of turnover in fast-twitch plantaris. These observations highlight the need to study turnover rates on a protein-by-protein basis and avoid generalisation of protein FSR data across muscles that have different protein compositions.

Investigations on muscle protein turnover have traditionally used radio- or stable- isotope-labelled amino acids and generated data by gas chromatography mass spectrometry of hydrolysates of free- and protein-bound tracer [26]. Such studies provide information on the average rate of turnover of all proteins in a muscle or sub-cellular fraction, and the methods can be adapted to target individual high-abundance proteins. Typically, the period of biosynthetic labelling is kept short (e.g., <30 min in laboratory rodents) to avoid recycling of label through the protein pool, which could confound the calculation of FSR. Protein FSR is calculated from the ratio between the amount of tracer measured in the protein-bound (product) and free amino acid (precursor) pool over the time period of the investigation. Based on a 10 min administration of radio-isotope-labelled phenylalanine in the rat [9], the average turnover of protein in soleus (9.6%/day ± 0.6%/day) was calculated to be approximately double that in fast-twitch tibialis anterior 4.5%/day ± 0.3%/day. These FSR values equate to an approximate protein half-life (t_1/2_) of 7 days in soleus and 14 days in tibialis anterior. Using 30 days D_2_O labelling, we report the average turnover of protein was ~2.26%/day (t_1/2_ = 27 days) in both soleus and plantaris. The apparent disparity in findings between earlier work [9] and our current data is probably explained by differences in the labelling period (10 min versus 30 days) and method of FSR calculation (linear versus non-linear) as well as the proportion of the proteome (mixture of all proteins versus a selection of individual proteins) studied. 

Differences in turnover rate between slow and fast-twitch muscles may be underpinned by muscle-specific protein compositions, particularly fast and slow isoforms of myofibrillar proteins. We report that individual proteins exhibit a broad range of turnover rates within skeletal muscle, but our analysis primarily focuses on proteins that are common to both soleus and plantaris muscle. To date, at least 17 papers (Table 2) have reported protein-specific FSR data in various muscles of humans [16,19,27,28], rodents [13,18,20,23,24,29,30,31,32,33,34,35] and chickens [17] in vivo. The earliest works (e.g., [16,23,30]) used biochemical techniques to isolate abundant individual proteins. For example, targeted analysis [30] of MyHC and muscle creatine kinase (KCRM) in rat abdominal muscle reported half-lives of 54.2 and 10.4 days, respectively, calculated using a 24 h infusion of stable isotope-labelled leucine. In our current work, two isoforms of creatine kinase were detected in plantaris and soleus. Mitochondrial creatine kinase (KCRS) is involved in intramitochondrial resynthesis of phosphocreatine by oxidative phosphorylation [36] and exhibited a significantly greater rate of turnover in plantaris than soleus. Whereas, the muscle isoform of creatine kinase (KCRM), which is a component of the sarcomeric M-band and catalyses extramitochondrial resynthesis of ATP, exhibited no significant difference turnover between soleus and plantaris. Regardless of the muscle studied, the turnover of KCRS was greater than KCRM (Table 1). 

Proteomic analysis of rat quadriceps muscle [29] provided protein-specific FSR data for 91 proteins using a 20 min infusion of an amino acid tracer. Protein FSR ranged from 0.16%/h ± 0.04%/h for MyHC to 1.5%/h ± 0.42%/h for dihydrolipoamine branched chain transacylase E2 [29]. If extrapolated to 24 h, the data reported in Jaleel et al. [29] equate to FSR values ranging from 3.84%/day to 36%/day and half-lives of 18 days to 2 days, which differ from our current findings (Table 1). This dissimilarity may be due to methodological differences in addition to differences in the muscles studied. The precursor: product calculation, used to calculate FSR in short-duration amino acid tracer studies assumes a linear relationship between protein turnover and the rate of accumulation of protein-bound tracers. In contrast, longer duration biosynthetic labelling experiments must account for the probability that label will be lost from the protein-bound pool due to the degradation of protein over the course of the experimental period. Assuming protein turnover is constant, the incorporation of label into the protein pool follows a non-linear exponential rise-to-plateau [21]. During the first few minutes of tracer infusion, the incorporation of label into the protein pool is likely to be essentially linear but extrapolation of FSR values from short-term (e.g., 10–20 min) biosynthetic labelling to longer periods (i.e., %/day values) will lead to overestimation of turnover rates [37]. Ten proteins were common between our work and data reported in Jaleel et al. [29]. On average, protein FSR (%/day) was 6.8-fold greater in Jaleel et al. [29], and the difference in protein-specific FSR ranged from 2.2-fold greater (KCRS) to 24-fold greater (Parvalbumin). 

The pattern of differences in FSR between soleus and plantaris (Table 1) is in agreement with our [18] report on the synthesis of eight proteins across four striated muscles (heart, diaphragm, EDL and soleus) in rats. In our earlier work, muscle proteins were resolved by 2-dimensional electrophoresis and matrix-assisted laser desorption ionisation (MALDI) mass spectrometry was used to collect peptide mass spectra. After 14 days of D_2_O labelling in vivo approximately 7% of beta-enolase (ENOB) and 3% of KCRM was newly synthesised in EDL, whereas 15% of ENOB and 9.5% of KCRM was newly synthesised in soleus. Such data on the relative proportion of D2O incorporation into protein after a specified labelling period (e.g., [18,31]), can be challenging to compare across studies. Whereas rate constants allow data to be compared across studies of differing durations. In EDL, the estimated fractional synthesis rate was 2.9%/day for ENOB and 8.3%/day for KCRM [18]. In the current plantaris data, FSR of ENOB was 1.7%/day and KCRM 2.09%/day. The lower values reported here in the plantaris muscle may be due to differences in muscle investigated (i.e., EDL vs. plantaris) or differences in the age of the animals (rats in Hesketh et al. [18] were ~100 g lighter than animals used in the current work). During the revision of this manuscript, Kallabis et al. [38] reported ^13^C_6_-lysine incorporation in muscle proteins in vivo combined with proteomic analysis of single fibres from mouse hindlimb muscles. The incorporation of ^13^C_6_-lysine was reported for 1720 proteins in type I, IIa, IIx and IIb fibres extracted from EDL, soleus, tibialis anterior and gastrocnemius muscles [38]. Such data are appropriate for within-muscle comparison of relative protein turnover, but the level of precursor enrichment was not measured, therefore synthesis data cannot be reported in FSR (%/day) units and protein half-life cannot be calculated. Nevertheless, there is an agreement between our analysis of protein FSR in rat and protein ^13^C_6_-lysine incorporation in mice. For example, Kallabis et al. [38] reported ^13^C_6_-lysine incorporation in N-myc downstream-regulated gene 2 protein (NDRG2) was particularly high, which is consistent with our finding that NDRG2 had the highest FSR amongst proteins measured in soleus.

**Figure 6:**
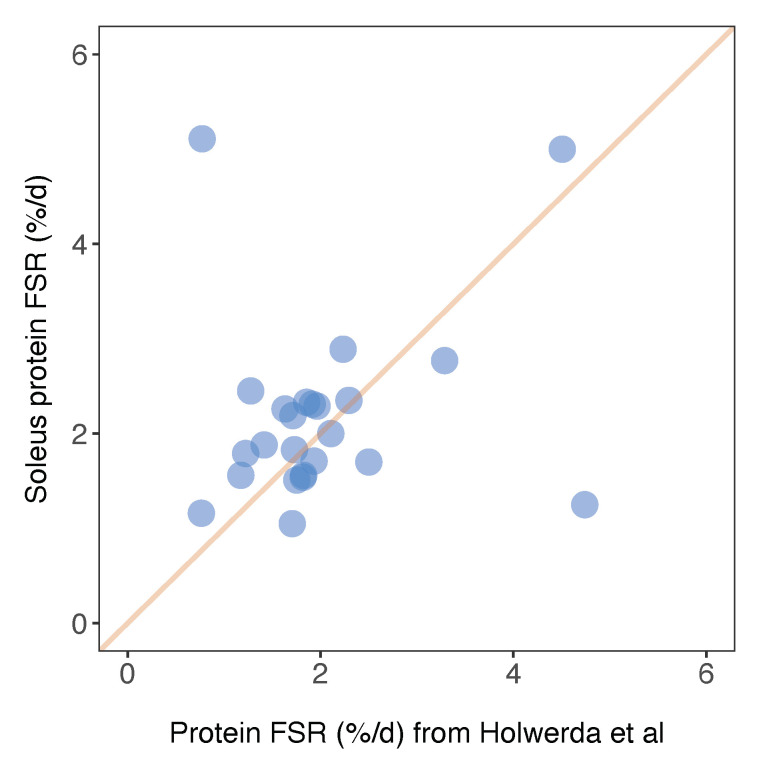
Comparison of soleus protein FSR data against published work. Protein-specific fractional synthesis rates in soleus muscle of Wistar rats reported in the current work (*y*-axis) plotted against equivalent data from soleus muscle of Lewis rats reported in Holwerda et al. [20]. Data represent *n* = 24 proteins that were common between the datasets, and a line of identity is included for comparison.

Herein, we report FSR data in %/day units that can be compared between animals and across studies. Similarly, Holwerda et al. [20] report FSR data in rat soleus, including 24 proteins that were also included in the current dataset. The factional turnover rate was remarkably similar for the majority of proteins (Figure 6), and the mean difference in FSR between the two studies was 0.15%/day. A small number of proteins exhibited a greater variance in turnover rates between the two studies (Figure 6). For example, we report serotransferrin (TRFE) turnover of 5.11%/day whereas Holwerda et al. report a >6-fold lesser rate (0.77%/day). Conversely, turnover of haemoglobin subunit beta 2 (HBB2) was 1.25%/day ± 0.14%/day in the current work and 4.74%/day ± 0.7%/day in [20]. FSR data exhibits greater biological variability than protein abundance data [19] but these inter-study differences may also relate to differences in rat strain or the analytical method used to calculate FSR. Holwerda et al. [20], employed a non-linear calculation consistent with the two-point model (Figure 3) that uses data collected at the start and end of the labelling period only. The calculation of synthesis from two data points assumes all proteins adhere to the expected exponential rise-to-plateau kinetics of deuterium incorporation. In the current work, we measured the incorporation of deuterium in proteins at four points during the course of the 30-day experimental period. Our results were filtered to exclude proteins that did not fit the expected exponential pattern (R^2^ of curve fitting must be >0.85), which removed ~10% of peptides and reduced the coefficient of variation amongst peptides belonging to the same protein. We believe this quality control step adds further confidence to our current data.

Three proteins: albumin (ALBU), carbonic anhydrase 3 (CAH3) and essential myosin light chain (MYL3) were common to soleus and plantaris and did not exhibit muscle-specific differences in FSR (Figure 5 and Table 1). ALBU is a prominent blood protein that is abundant in muscle and responsible for interstitial fatty acid transportation [39]. The rate of turnover of ALBU reported here (~5%/day) is consistent with ~5.6%/day reported in four striated muscles of the rat in our earlier work [18]. The close similarity in ALBU turnover rate across different muscles is consistent with a single common origin of ALBU from the liver rather than site-specific synthesis of ALBU in each muscle. CAH3 may account for ~10% of the soluble protein fraction in skeletal muscle [40] and the turnover of CAH3 is reported [24] to reflect the global turnover rate of skeletal muscle. We report the rate of CAH3 turnover is indistinguishable between soleus and plantaris (Figure 2) but also that the CAH3 turnover rate differs from the turnover of other individual proteins studied. MYL3 is the slow isoform of myosin essential light chain and was detected in both soleus and plantaris, whereas the fast isoform, MYL1, was detected in plantaris only. These findings differ from our previous work [18], which suggested inter-muscle differences in MYL3 turnover based on the proportion of newly synthesised protein after a fixed period of D_2_O administration. 

We report novel data on the FSR of 44 proteins in soleus and 34 proteins in plantaris of rats using D_2_O labelling in vivo and peptide mass spectrometry. Our findings are consistent with the limited equivalent published data on protein-specific FSR but differ from mixed protein studies that found clear differences in the average rate of protein turnover between slow and fast skeletal muscles. Our current analysis is limited to a relatively small number of proteins. It could be assumed that if individual synthesis rates for a greater proportion of the proteome were included, the differences between the two muscle phenotypes would emerge. It remains to be shown whether expected differences between the relative abundance of proteins in soleus and plantaris may associate with our reported differences in turnover rate. Protein attributes, including abundance, sequence motifs and sub-cellular location, have been shown to be relatively weak predictors of protein-specific FSR in model systems [11] but have not yet been extensively studied in animals in vivo. 

In summary, protein-specific FSR data from different muscles cannot be used interchangeably. This study, and previous work from our lab [18], using D_2_O labelling in vivo, emphasises that the turnover rates of individual proteins are specific to different striated muscles. Longer established measurements of mixed protein synthesis using stable isotope-labelled amino acids offer an overview of the average rate of turnover of muscle proteins but do not readily allow for individual proteins to be investigated. Future research will benefit from measuring the rates of synthesis of individual proteins, particularly when investigating phenomena that are associated with changes in muscle protein composition.

## Figures and Tables

**Table 1:** Protein-specific fractional synthesis rates (FSR; %/day) in soleus and plantaris muscle.

Acc.	Description	Soleus	Plantaris	*p* Value	BH
ADT1	ADP/ATP translocase 1	2.65 ± 0.075	2.83 ± 0.142	0.1512	0.2403
ALBU	Albumin	5.01 ± 0.62	5.21 ± 3.82	0.6379	0.7385
ALDOA	Fructose bisphosphate aldolase α	2.89 ± 1.106	2.49 ± 0.12	0.5682	0.7232
ATPA	ATP synthase α	2.33 ± 0.378	2.77 ± 0.197	0.1455	0.2264
ATPB	ATP synthase β	2.52 ± 0.257	2.98 ± 0.094	0.0416	0.0832
CAH3	Carbonic anhydrase 3	1.71 ± 0.389	1.77 ± 0.208	0.8376	0.9020
ENOB	β-enolase	2.45 ± 0.352	1.69 ± 0.053	0.0207	0.0579
G3P	Glyceraldehyde-3-phosphate dehydrogenase	2.19 ± 0.167	1.82 ± 0.082	0.0271	0.0632
HBB1	Haemoglobin β-1	1.49 ± 0.119	1.95 ± 0.163	0.0166	0.0579
KAD1	Adenylate kinase isoenzyme 1	1.56 ± 0.66	2.46 ± 0.629	0.2232	0.3153
KCRM	Creatine kinase M-type	2.26 ± 0.372	2.09 ± 0.039	0.4734	0.6628
KCRS	Creatine kinase S-type	2.52 ± 0.375	4.99 ± 0.669	0.0050	0.0349
KPYM	Pyruvate Kinase	1.7 ± 0.246	2.61 ± 0.099	0.0039	0.0349
MDHM	Malate dehydrogenase, mitochondrial	2.31 ± 0.341	2.41 ± 0.078	0.6253	0.7295
MYG	Myoglobin	1.54 ± 0.152	2.03 ± 0.077	0.0075	0.0349
MYL3	Myosin essential light chain, slow/ventricular	2.26 ± 0.255	2.26 ± 0.088	0.9670	0.9670
PGAM2	Phosphoglycerate mutase 2	1.79 ± 0.199	2.07 ± 0.146	0.1602	0.2403
PGM1	Phosphoglucomutase-1	1.05 ± 0.051	2.4 ± 0.328	0.0118	0.0531
TPIS	Triosephosphate isomerase	1.88 ± 0.232	1.41 ± 0.194	0.0535	0.0936
AATC	Aspartate aminotransferase, cytoplasmic	2 ± 0.438	-	-	-
AATM	Aspartate aminotransferase, mitochondrial	2.27 ± 0.216	-	-	-
ACON	Aconitate hydratase, mitochondrial	2.08 ± 0.043	-	-	-
ACTS	Actin, α skeletal muscle	1.16 ± 0.123	-	-	-
AT2A1	Sarcoplasmic/endoplasmic reticulum calcium ATPase 1	-	3.42 ± 0.271	-	-
AT2A2	Sarcoplasmic/endoplasmic reticulum calcium ATPase 2	3.64 ± 0.539	-	-	-
CASQ1	Calsequestrin 1	-	0.81 ± 0.068	-	-
CO1A1	Collagen α-1 (I) chain	0.58 ± 0.16	-	-	-
COF1	Cofilin-1	2.86 ± 0.131	-	-	-
CRYAB	α-crystallin B chain	3.6 ± 0.349	-	-	-
CS044	Uncharacterized protein C19orf44 homolog	1.13 ± 0.196	-	-	-
ETFA	Electron transfer flavoprotein subunit alpha, mitochondrial	2.35 ± 0.345	-	-	-
FABPH	Fatty acid-binding protein, heart	1.96 ± 0.333	-	-	-
FHL1	Four and a half LIM domains protein 1	2.78 ± 0.463	-	-	-
FLNC	Filamin-C	3.13 ± 0.411	-	-	-
G6PI	Glucose-6-phosphate isomerase	-	2.32 ± 0.836	-	-
H2B1	Histone H2B type 1-α	-	0.77 ± 0.099	-	-
HBA	Haemoglobin subunit α-1/2	1.51 ± 0.095	-	-	-
HBB2	Haemoglobin subunit β-2	1.25 ± 0.143	-	-	-
HSP7C	Heat shock cognate 71 kDa protein	2.77 ± 0.659	-	-	-
HSPB1	Heat shock protein β-1	3.62 ± 0.38	-	-	-
IDHP	Isocitrate dehydrogenase [NADP], mitochondrial	2.62 ± 0.267	-	-	-
LDHA	Lactate dehydrogenase α chain	-	2.49 ± 0.55	-	-
LDHB	Lactate dehydrogenase β chain	3.12 ± 0.405	-	-	-
MDHC	Malate dehydrogenase, cytoplasmic	2.29 ± 0.385	-	-	-
MLRS	Myosin regulatory light chain 2, skeletal muscle	-	1.65 ± 0.343	-	-
MLRV	Myosin regulatory light chain 2, ventricular/cardiac muscle isoform	1.36 ± 0.3	-	-	-
MYH4	Myosin heavy chain 4	-	2.27 ± 0.183	-	-
MYH8	Myosin heavy chain 8	-	2.38 ± 0.14	-	-
MYL1	Myosin essential light chain, fast/skeletal muscle	-	1.64 ± 0.013	-	-
NDRG2	Protein NDRG2	5.4 ± 0.588	-	-	-
PEBP1	Phosphatidylethanolamine-binding protein 1	2.05 ± 0.036	-	-	-
PGK1	Phosphoglycerate kinase 1	1.56 ± 0.314	-	-	-
PRVA	Parvalbumin α	1.31 ± 0.019	-	-	-
PYGB	Glycogen phosphorylase, brain form	-	3.04 ± 0.256	-	-
PYGM	Glycogen phosphorylase, muscle form	-	2.86 ± 0.072	-	-
SODC	Superoxide dismutase [Cu-Zn]	1.83 ± 0.225	-	-	-
TNNT3	Troponin T, fast skeletal muscle	-	3.24 ± 0.162	-	-
TPM1	Tropomyosin α-1 chain	-	1.91 ± 0.196	-	-
TPM2	Tropomyosin β chain	-	1.7 ± 0.071	-	-
TRFE	Serotransferrin	5.11 ± 0.57	-	-	-

Fractional synthesis rates (FSR) expressed as %/day and presented as mean ± SD of *n* = 3 biological replicates. Paired *t*-tests of each biological replicate (*n* = 3) for each protein were used to identify statistical differences in FSR illustrated in Figure 5.

**Table 2:** Summary of literature reporting targeted or omic analysis of protein-specific fractional synthesis rates (FSR) in skeletal muscle using stable isotope labelling in vivo.

Citation	Organism: Muscle (*n*)	Stable Isotope Label (Duration, Route)	Exp Type (Number of Proteins)
Hasten et al., 1998. [16]	Human: Vastus lateralis (6)	[1-^13^C]-Leucine (14 h i.v. infusion)	Targeted (2)
Papageorgopoulos et al., 2002. [30]	Rat: Hindlimb leg and heart (2)	[5,5,5-^2^H_3_]-Leucine (24 h i.v. infusion)	Targeted (2)
Doherty et al., 2005. [17]	Chicken: Pectoralis (3)	[^2^H_8_]-valine (5 d in diet)	Omic (8)
Jaleel et al., 2008. [29]	Rat: Quadriceps (6)	[^13^C_6_]-phenylalanine (15 min i.v. bolus)	Omic (91)
Claydon et al., 2012. [32]	Mice: Heart and hindlimb (2)	[^2^H_8_]-valine (12 d in diet)	Omic (56)
Scalzo et al., 2014. [28]	Human: Vastus lateralis (22)	D_2_O (28 d drinking water)	Omic (381)
Karunadharma et al., 2015. [33]	Mouse: Mitochondrial enriched fraction of Heart, Liver, Brain, Soleus and EDL (4)	[5,5,5-^2^H_3_]-Leucine (17 d in diet)	Omic (84)
Hammond et al., 2016. [13]	Bank Vole: Heart, kidney, liver and hindlimb (2)	[^13^C_6_]-lysine (1, 5, 12, 25 and 40 d in diet)	Omic (358)
Shankaran et al., 2016. [24]	Rat: Gastroc (4) Human: Quadriceps (2-11)	D_2_O (4 d drinking water) D_2_O (21 d drinking water)	Omic (75) Omic (273)
Shankaran et al., 2016 [31]	Rat: Triceps, EDL, Soleus. (3-5)	D_2_O (4, 5 and 8 d drinking water)	Omic (125)
Hesketh et al., 2016. [18]	Rat: Heart, diaphragm, EDL and soleus (3)	D_2_O (14 d drinking water)	Omic (8)
Kruse et al., 2016. [34]	Mouse: Mitochondria enriched fraction of Soleus and EDL (8)	[5,5,5-^2^H_3_]-Leucine (28 d in diet)	Omic (745)
Camera et al., 2017. [14]	Human: Vastus lateralis (8)	D_2_O (9 d drinking water)	Omic (91)
Murphy et al., 2018. [27]	Human: Vastus lateralis (10)	D_2_O (28 d drinking water)	Omic (190)
Srisawat et al., 2019. [19]	Human: Vastus lateralis (4)	D_2_O (14 d drinking water)	Omic (54)
Holwerda et al., 2020. [20]	Rat: Soleus (3)	D_2_O (21 d drinking water)	Omic (108)
Miller et al., 2020. [35]	Mouse: Quadriceps, Heart, Liver, White adipose tissue (5-10)	D_2_O (14 d drinking water)	Omic (31)

## Supplementary Materials

The following are available online at https://www.mdpi.com/2227-7382/8/2/10/s1. Supplementary Table S1 reports peptide FSR data per animal in machine-readable format and other supporting data, including peptide mass isotopomer abundance, etc. are available upon request.
